# Observational Multicenter Study on the Prognostic Relevance of Coagulation Activation in Risk Assessment and Stratification in Locally Advanced Breast Cancer. Outline of the ARIAS Trial

**DOI:** 10.3390/cancers12040849

**Published:** 2020-04-01

**Authors:** Laura Pizzuti, Eriseld Krasniqi, Chiara Mandoj, Daniele Marinelli, Domenico Sergi, Elisabetta Capomolla, Giancarlo Paoletti, Claudio Botti, Ramy Kayal, Francesca Romana Ferranti, Isabella Sperduti, Letizia Perracchio, Giuseppe Sanguineti, Paolo Marchetti, Gennaro Ciliberto, Giacomo Barchiesi, Marco Mazzotta, Maddalena Barba, Laura Conti, Patrizia Vici

**Affiliations:** 1 Division of Medical Oncology 2, IRCCS Regina Elena National Cancer Institute, Via Elio Chianesi, 00144 Rome, Italy; laura.pizzuti@ifo.gov.it (L.P.); krasniqier@gmail.com (E.K.); domenico.sergi@ifo.gov.it (D.S.); elisabetta.capomolla@ifo.gov.it (E.C.); giancarlo.paoletti@ifo.gov.it (G.P.); patrizia.vici@ifo.gov.it (P.V.); 2 Department of Clinical Pathology, IRCCS Regina Elena National Cancer Institute, Via Elio Chianesi 53, 00144 Rome, Italy; chiara.mandoj@ifo.gov.it (C.M.); laura.conti@ifo.gov.it (L.C.); 3 Medical Oncology Unit, Sant’Andrea Hospital, Via di Grottarossa, 1035/1039, 00189 Rome, Italy; danielemarinelli1@gmail.com (D.M.); paolo.marchetti@uniroma1.it (P.M.); 4 Department of Surgery, IRCCS Regina Elena National Cancer Institute, Via Elio Chianesi 53, 00144 Rome, Italy; claudio.botti@ifo.gov.it; 5 Radiology Department, IRCCS Regina Elena National Cancer Institute, Via Elio Chianesi 53, 00144 Rome, Italy; ramy.kayal@ifo.gov.it (R.K.); francescaromana.ferranti@ifo.gov.it (F.R.F.); 6 Biostatistics Unit, IRCCS Regina Elena National Cancer Institute, Via Elio Chianesi 53, 00144 Rome, Italy; isabella.sperduti@ifo.gov.it; 7 Pathology Department, IRCCS Regina Elena National Cancer Institute, Via Elio Chianesi 53, 00144 Rome, Italy; letizia.perracchio@ifo.gov.it; 8 Department of Radiation Oncology, IRCCS Regina Elena National Cancer Institute, Via Elio Chianesi 53, 00144 Rome, Italy; giuseppe.sanguineti@ifo.gov.it; 9 Scientific Direction, IRCCS Regina Elena National Cancer Institute, Via Elio Chianesi 53, 00144 Rome, Italy; gennaro.ciliberto@ifo.gov.it; 10 Medical Oncology Unit, Ospedale dell’Angelo, Via Paccagnella 11, 30174 Mestre, Italy; giacomo.barchiesi88@gmail.com

**Keywords:** coagulation activation, locally advanced breast cancer, prognostic model, pCR, venous thromboembolism

## Abstract

A hypercoagulable state may either underlie or frankly accompany cancer disease at its onset or emerge in course of cancer development. Whichever the case, hypercoagulation may severely limit administration of cancer therapies, impose integrative supporting treatments and finally have an impact on prognosis. Within a flourishing research pipeline, a recent study of stage I-IIA breast cancer patients has allowed the development of a prognostic model including biomarkers of coagulation activation, which efficiently stratified prognosis of patients in the study cohort. We are now validating our risk assessment tool in an independent cohort of 108 patients with locally advanced breast cancer with indication to neo-adjuvant therapy followed by breast surgery. Within this study population, we will use our tool for risk assessment and stratification in reference to 1. pathologic complete response rate at definitive surgery, intended as our primary endpoint, and 2. rate of thromboembolic events, intended as our secondary endpoint. Patients’ screening and enrollment procedures are currently in place. The trial will be shortly enriched by experimental tasks centered on next-generation sequencing techniques for identifying additional molecular targets of treatments which may integrate current standards of therapy in high-risk patients.

## 1. Introduction

Large and consistent evidence supports the mutual association between cancer and coagulation abnormalities, with cancer being increasingly renowned as a predisposing factor for thromboembolic events [1]. Indeed, at the general population level, incidence rates for venous thromboembolism (VTE) approximate one to two cases per 1000 people/year, while cancer patients generally show a 4 to 10 times greater risk [2]. Consistently, VTE is associated with poor prognosis of disease in glioblastoma, ovarian, colorectal, lung and pancreatic cancer [3,4,5]. In strict regard to breast cancer, data are available in support of the prognostic relevance of coagulation activation on survival in both advanced and early disease [6]. 

Our research team has long and actively participated in initiatives led by the Cochrane collaboration, a global network of health professionals [7], with a focus on the design and update of systematic reviews (SR) and meta-analyses (MA) of anticoagulants use in cancer patients. In reference to our findings from a quite recently published work, which included data for a total of 1486 participants, we could not elicit a mortality benefit from the use of anticoagulants in cancer patients. However, when addressing the overall completeness and applicability of our study results, we pointed out that the greatest majority of the data analyzed were from randomized trials of lung cancer patients. On this basis, we underlined the need for further ad hoc studies focused on the effects of anticoagulants in patients with different types and stages of cancer [8]. In this view, given a prespecified type of cancer and stage at diagnosis, further steps of a well-focused research strategy are ideally aimed at clarifying the extent to which patient- and cancer-related features, along with differences in the activation state of the coagulation cascade, may affect health outcomes in reference to both cancer- and VTE-related endpoints. 

Based on prior work performed in collaboration with the Cochrane network and supported by the expertise of scientists who have long operated in the management of thrombosis in cancer patients, we are now further extending our research pipeline on coagulation activation in breast cancer. We have previously moved our very first step within the early setting and carried out an observational study of 235 stage I-IIA patients with a 95-month follow up. Based on procedures whose details will be summarized in the methods section, we developed a prognostic model for the assessment and stratification of risk of death upon factors related to relevant clinic-pathologic characteristics and coagulation profile. This tool is shown in Table 1 and has proven efficacy in distinguishing patients’ categories characterized by significantly different survival estimates [9]. On this basis, more recently, we have required and obtained formal approvals from the dedicated institutional bodies for the conduct of a validation study on an independent cohort of breast cancer patients consenting to participate and to be prospectively followed in a trial on the prognostic relevance of biomarkers of coagulation activation for risk assessment and stratification in reference to patients’ important outcomes in breast cancer. This trial outline will be described across the following sections of the manuscript herein presented.

*Risk categories:*

*Score = 0-1 → High probability of* pathologic complete response (*pCR).*
*Score = 2* *→* *Intermediate probability of pCR.*
*Score > 2* *→* *Low probability of pCR.*

## 2. Results

Results available at the time of writing are preliminary only. The Institutional Review Board (IRB) of the coordinating center, the IRCCS Regina Elena National Cancer Institute, has released formal approval for the ARIAS trial in January 2020 (Register code: RS1307/19_2303). The documents considered for IRB approval included a written consent form for patients’ consultation and, eventually, signing prior to any study procedures. 

**Figure 1:**
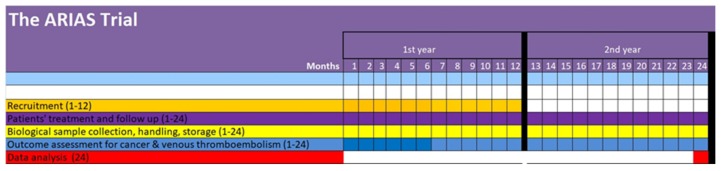
ARIAS Trial Gantt chart.

The study Gantt chart is displayed in Figure 1. Patients’ characteristics to be evaluated for study eligibility are summarized in the methods section. Thus far, within a time window of approximately 40 days, four patients have been identified as potentially suitable for inclusion based on the available clinical, instrumental and pathological records. The screened/enrolled ratio for patients’ participation is currently equal to 1, i.e., 3/3. In more detail, three patients were invited to adhere and undersigned the written consent form, while the fourth will be contacted at the time of completion of the diagnostic workup. 

For patients enrolled, data concerning the variables of interest have been collected in a face-to-face interview carried out by specifically trained medical personnel involved in this trial conduct. Blood sample collection was performed according to highly standardized operative procedures (SOPs) previously set by dedicated personnel of our clinical pathology lab. Similarly, SOPs have been codified concerning blood sample handling, storage and biomarkers assessment as reported in more detail in the methods section. Patients’ enrollment, data and baseline blood sample collection took place or will take place at the time of the first access to the oncology day hospital (DH) for chemotherapy administration. Thus far, no delays or, more generally, negative interferences were reported for the activities related to the therapeutic management of the patients enrolled. The ARIAS participants will be prospectively followed by dedicated personnel. Their profile will be updated in parallel with the DH accesses for chemotherapy administration. Blood sample collection will be repeated at the time of last chemotherapy, prior to breast surgery. Pathologic assessment of surgical breast samples will provide data related to the primary study endpoint, i.e., pathologic complete response (pCR) (yes/no). Data on thromboembolic events will be collected in course of therapy administration and following breast surgery, with the latest update being scheduled 6 months after surgery.

As specified in the Methods section, the ARIAS trial has been conceived as a multicentric initiative. Patients enrollment will shortly be active also at the four satellite centers. On average, based on the trial design, the estimated enrollment capacity of each of the centers involved is expected to broadly vary within a 1-to-3 patient/month range. Thus, the expected number of patients enrolled within a 12-month time window will be encompassed within a 60-to-180 range, which will fully address the minimum number of patients to be enrolled required based on the sample size calculations reported in the methods section. Over the course of the second year, therapy administration, biomarkers’ assessment and patients follow up will inevitably overlap and converge towards the study closure and data analysis. Overall, a study length of about 2 years is foreseen, with a potential no-cost extension of about 6 months.

## 3. Discussion

We have herein outlined the main features of the ARIAS trial, a multicenter, observational study with prospective design focused on the prognostic role of biomarkers of coagulation activation in breast cancer patients undergoing neoadjuvant chemotherapy (NACT) and breast surgery in clinical practice. This trial is well placed within a research pipeline on coagulation and cancer. It is primarily aimed at validating the prognostic accuracy of our previously developed prognostic model in an independent cohort of breast cancer patients [9]. The ARIAS trial has received formal approval by the IRB of the coordinating center. The enrollment procedures have started. Results obtained thus far in terms of recruitment rate and study feasibility are extremely encouraging.

Our prior work was funded on the hypothesis that the activation state of the coagulation cascade significantly concurs to the definition of patients’ important outcomes in breast cancer, along with patient- and cancer-related features. Breast cancer patients may thus be allocated to different risk categories for the outcomes of interest based on the use of the tool we developed. Upon validation, this prognostic model may help inform therapeutic decisions and favor the use of alternative or integrating treatments in patients for whom less favorable outcomes are foreseen. 

In our prior study, we identified patient- and disease-related characteristics with prognostic relevance and included them into a prognostic model which proved accuracy in risk assessment and stratification in reference to the survival outcomes assessed in a cohort of 235 breast cancer patients diagnosed and treated at the IRCCS Regina Elena National Cancer Institute (IRE) between 2008 and 2010 [9]. Age, pathologic T (pT), baseline circulating levels of D-dimer (DD) and factor VIII (FVIII) proved prognostically relevant in multivariate models of overall survival (OS). The identified outcome predictors were then used for prognostic score assessment. The score including these factors proved efficacious in distinguishing risk categories in reference to OS, in that patients within the lowest risk category showed significantly longer OS compared to their counterparts (*p* < 0.0001). Based on our prior experience, we now expect significantly less favorable outcomes in the highest category of risk compared to the lowest as defined based on the prognostic model for risk assessment and stratification in course of validation. In strict referral to the single outcomes and related endpoints, this may translate into lower pCR rates and higher rates of VTE-related events for patients placed within the highest risk category. While conducting the ARIAS trial, some problems may emerge. Concerning the recruitment target of at least 108 patients in about 12 months defined upon ad hoc calculations, at the coordinating center, the average number of patients with an indication to NACT followed by breast surgery is of about 1–3 per month. This would allow the recruitment of 12–36 patients in 12 months. The remaining patients will be recruited at the collaborating centers, which will support our project working in kind and whose recruitment capacities are overall fairly comparable to ours. 

Though statistically adequate in reference to our cancer-related endpoint, the sample size of our study population is relatively restricted. The overall number of deep vein thrombosis (DVT) and pulmonary embolism (PE) cases occurring within a 12-month time window in such a population may be relatively low. Indeed, based on literature data, the reported 1-year VTE incident rate (events per 100 patients per year) is about 0.9 [10]. However, literature data also support a relevant impact of several patient- and disease-related characteristics on VTE incident rates in cancer patients [11]. In more detail, VTE-related events are more often detected at the time of cancer diagnosis or within the first 6 months from diagnosis, as well as in patients with relevant disease burden, those with fast-growing tumors and those who receive anti-neoplastic agents [6,12,13,14]. In addition, an increased incidence of VTE is associated with the use of indwelling upper extremity and central venous catheters (CVC), with the incidence rates of clinically overt CVC-related DVT in cancer patients potentially rising to 28.3% [15]. All the previously mentioned patient- and disease-related features were considered at the time of our study conception and will be represented within the ARIAS cohort. This may considerably increase the VTE incident rates observed in the study under consideration. However, at the study closure, we intend to perform ad hoc power calculation to estimate the ARIAS power in reference to the VTE endpoint in light of our accomplishment in terms of number of patients enrolled and VTE events that occurred. It is also noteworthy to highlight the different stage of development of the prognostic model in reference to our primary and secondary endpoint. Indeed, the ARIAS was conceived with validation purposes in reference to our primary endpoint, i.e., pCR. Conversely, no VTE-related endpoints have been addressed in our prior study [9]. Thus, data from the ARIAS will be used to identify prognostically relevant factors in reference to VTE outcomes. This suggests the need of further validation of our model in reference to VTE outcomes in an independent cohort. 

As previously stated, the ARIAS trial is intended as a validation study in reference to our primary endpoint, i.e., pCR rate following definitive breast surgery. In orienting our choice, we considered data from consistent literature in support of the predictive role of pCR on survival outcomes, with more favorable event-free and overall survival, particularly in patients diagnosed with the most aggressive breast cancer subtype [16,17]. These data support the correct use of pCR as a short-term surrogate of survival, offering the possibility to validate our previous risk model built on survival outcomes within a short follow-up framework. However, we are committed to also report on the accuracy of our model in predicting survival outcomes for the ARIAS participants when data from an adequately long follow up will be available. In these respects, an appropriate median length of follow up may be set at three years [16]. 

In the ARIAS trial, strengths and potentials for innovation come from the following key points: 1. the evidence elicited in our prior study in support of the role of coagulation activation in cancer will be now be validated in a study population which is homogeneous by primitive cancer site and setting at diagnosis; 2. The predefined sample size and prospective design confer an acceptable quality to our data. In addition, we are planning additional experimental tasks which will allow for the assessment of the burden of mutational events throughout a next-generation sequencing (NGS) approach in paraffin-embedded, formalin-fixed (PEFF) breast tissue samples collected at baseline. The presence of a higher mutational burden assessed by NGS in the highest risk category may further confirm our model in terms of accuracy. 

## 4. Methods and Statistical Analysis

The ARIAS trial was conceived as a validation trial focused on a prognostic model developed in our prior study [9]. The tool we developed included patient- and disease-related features along with biomarkers of coagulation activation. In the herein proposed study, clinical–pathological data will be collected at baseline. Two tubes for citrate vacutainer and 3 EDTA will be collected at study entrance and before surgery, then centrifuged, aliquoted and stored at -80 °C for subsequent tests. The following components of the hemostatic systems will be immediately analyzed by coagulation, chromogenic and immunological methods on a fully automated ACLTOP analyzer using commercial HemosIL® kits (Instrumentation Laboratory Company, Bedford, MA USA): PT, aPTT, Resistance (APCR) Fibrinogen, FVIII, DD, Protein C (PC), Protein S (PS), Antithrombin (AT) and Activated Protein C. 

The accuracy of our prognostic model will be validated against the following endpoints: 1. Cancer-related endpoints, exemplified in this setting by pCR, defined as no residual invasive cancer, both in breast and axilla, i.e., ypT0/is ypN0. The assessment will be performed in tissue samples collected at the time of definitive surgery. Breast surgery will be performed within a time window of about 4 weeks from the end of the last cycle of NACT. 2. VTE-related endpoints, exemplified by symptomatic deep vein thrombosis (DVT), symptomatic pulmonary embolism (PE), central vein catheter (CVC) thrombosis and arm vein thrombosis. DVT will be confirmed by 1. compression ultrasonography (US) showing new or prior undocumented non-compressibility of one or more proximal venous segments, popliteal or higher of the legs; or 2. venography showing constant intraluminal filling defect in two or more vessels. PE will be confirmed by 1. spiral computed tomography (CT) identifying thrombus in pulmonary vessels or 2. ventilation/perfusion lung scan showing one or more perfusion mismatches. Arm vein thrombosis and CVC thrombosis will be assessed based on DD testing and US.

The ARIAS trial is being conducted in accordance with the Declaration of Helsinki. 

Eligible participants are stage IIB-IIIC breast cancer patients undergoing NACT followed by breast surgery according to the current guidelines. The following inclusion criteria are to be met: age older than 18 years, normal range of kidney and liver function parameters, ECOG performance status ≤2, and a baseline left ventricular ejection fraction of at least 55 percent. At enrollment, participants must have no prophylactic or therapeutic indications to the use of anticoagulants. Reasons for exclusions are pregnancy, metastatic breast cancer, previous chemotherapy, prior hormonal therapy, prior radiotherapy, prior malignancies or contralateral breast cancer. 

Study participants will be followed up at the Institute of reference over the course of NACT until breast surgery. Pathologic assessment of surgical breast tissues is planned to be performed in loco, with a central revision in a randomly selected 10% of the samples analyzed at the satellite institutes. Similarly, a second radiologist will perform an independent review of the ultrasound (US) and CT images for 10% of the cases assessed in reference to VTE-related endpoints. Biomarkers of coagulation activation will be centrally assessed. 

Descriptive statistics will be computed for all the variables of interest. Means and standard deviations will be used to describe continuous variables. Categorical variables will be addressed by χ2 test or Fisher’s exact test. A receiver operating curve (ROC) approach will be used to evaluate the accuracy of the prognostic model in reference to pCR outcomes. VTE data will be analyzed according to a time-to-event approach using the product limit estimator to build Kaplan Meier survival curves according to the variables of interest and compare them by log-rank test. Cox regression models will be used to compute the effect of multiple factors on the VTE risk, and identify those relevant. The Harrell’s concordance index will be used to calculate the risk scores. Statistical analysis will be performed by using the SPSS and MedCalc software.

## 5. Sample Size Calculations

The primary objective is the validation of the accuracy of the prognostic model developed in our prior study and inclusive of clinical–pathological factors, i.e., clinical T (cT), D Dimer and Factor VIII, in patients with locally advanced stage IIB-IIIC breast cancer, treated with NACT and breast surgery in clinical practice. To the purpose of our trial, we will enroll at least 108 patients with the previously defined characteristic features. This sample size has been defined by considering an AUC value of 0.95 as significant compared to a value of 0.84 (null hypothesis) and a low/high risk ratio, i.e., better/worse prognosis, equal to 2, at a level of significance or 0.05 and with a statistical power of 80%.

## 6. Conclusions

In summary, we have briefly provided the outline of the ARIAS trial, a multicenter, observational trial with prospective design, which has recently been granted with IRB approval for the coordinating center and has thus started the enrollment procedures with encouraging results. The ARIAS trial is a validation study in referral to our primary endpoint. As such, it is placed within a pre-existing research pipeline centered on the key role of coagulation activation in cancer, with a specific focus on breast cancer. The evidence we are currently producing throughout this trial conduct hold acceptable quality, since it stems from a study conducted with a prospective design, in which ad hoc sample size calculations were performed and study endpoints predefined. In addition, SOPs were applied to the collection, handling and assessment of the biomarkers of interest. Our study potentials will be shortly further enriched by additional tasks focused on the NGS assessment of key genes of relevant pathways related to coagulation activation as assessed in PEFF bioptic tissue samples collected at baseline.

## Figures and Tables

**Table 1:** Prognostic score assessment in the ARIAS trial participants.

Score Factors	Score Points		
	0	1	2
**pT**	T2	–	T3-4
**FVIII**	Normal	Abnormal	–
**Age**	≤ 70	> 70	–
**DD**	Low	–	High

pT, pathologic; T, Age in years; FVIII, factor VIII; normal range for the lab of reference, 50–150%; *D,* d-dimer; normal values for the lab of reference < 280 ng/mL.
